# Analysis of traumatic event emergency department visits among care home residents aged 65 + years in Southern Jutland, Denmark: implications for comprehensive care and subsequent hospital admissions - a register-based cohort study

**DOI:** 10.1186/s12877-024-05092-0

**Published:** 2024-05-28

**Authors:** Zuhreh Sarwari, Gitte Schultz Kristensen, Sofie Ronja Petersen, Christian Backer Mogensen

**Affiliations:** 1 Department of Clinical Research, University Hospital of Southern Denmark, Aabenraa, Denmark; 2 Emergency Department, Aabenraa Hospital, University Hospital of Southern Jutland, Aabenraa, Denmark; 3 Department of Regional Health Research, Faculty of Health Science, University of Southern Denmark, Odense, Denmark; 4 Department of Clinical Research and Emergency Department, Aabenraa Hospital, University Hospital of Southern Denmark, Aabenraa, Denmark

**Keywords:** Treat and release, Traumatic events, Injury, Casualty, Emergency department, Acute admission, Nursing home, Care home, Older adults, Register-based

## Abstract

**Background:**

Care home residents aged 65 + years frequently experience acute health issues, leading to emergency department visits. Falls and associated injuries are a common cause of these visits and falls in a geriatric population can be a symptom of an incipient acute illness such as infection. Conversely, the traumatic event can cause illnesses to arise due to consequences of the fall, e.g. delirium or constipation due to opioid use. We hypothesised that a traumatic event treat-and-release emergency department visit serves as an indicator for an upcoming acute hospital admission due to non-trauma-related conditions.

**Methods:**

We studied emergency department visits for traumatic events among all care home residents aged 65+ (*n* = 2601) living in Southern Jutland, Denmark, from 2018 to 2019. Data from highly valid national registers were used to evaluate diagnoses, mortality, and admissions. Cox Regression was used to analyse the hazard of acute hospital admission following an emergency department treat-and-release visit.

**Results:**

Most visits occurred on weekdays and during day shifts, and 72.0% were treated and released within 6 h. Contusions, open wounds, and femur fractures were the most common discharge diagnoses, accounting for 53.3% of all cases (*n* = 703). In-hospital mortality was 2.3%, and 30-day mortality was 10.4%. Among treat-and-release visits (*n* = 506), 25% resulted in a new hospital referral within 30 days, hereof 13% treat-and-release revisits (duration ≤ 6 h), and 12% hospital admissions (duration > 6 h). Over half (56%) of new hospital referrals were initiated within the first seven days of discharge. Almost three-fourths of subsequent admissions were caused by various diseases. The hazard ratio of acute hospital admissions was 2.20 (95% CI: 1.52–3.17) among residents with a recent traumatic event treat-and-release visit compared to residents with no recent traumatic event treat-and-release visit.

**Conclusion:**

Traumatic event treat-and-release visits among care home residents serve as an indicator for subsequent hospitalisations, highlighting the need for a more comprehensive evaluation, even for minor injuries. These findings have implications for improving care, continuity, and resource utilisation.

**Trial registration:**

Not relevant.

**Supplementary Information:**

The online version contains supplementary material available at 10.1186/s12877-024-05092-0.

## Background

Emergency department (ED) visits are common among older adults in care homes [1]. ED visits can result from traumatic events, acute medical conditions, or deterioration of chronic diseases. Care home residents (CHRs) are more often admitted acutely to ED compared to their community-dwelling peers [2, 3] and have a higher risk of experiencing in-hospital adverse events [4, 5]. Understanding the causes and extent of ED visits among CHRs is crucial for improving care, preventing unnecessary hospitalisations, and optimising resource utilisation in the healthcare system.

Studies show that falls and fall-related presentations account for around a quarter of all ED visits by CHRs [4, 6–8] and more than half (59%) of all outpatient ED visits [9]. One-third of all adults over 65 fall at least once every year [10], and ground-level falls among older adults are associated with significant morbidity and mortality [11]. Both advanced age and multi-morbidity increase the risk of falling [12–14], giving the CHRs an even higher risk of experiencing falls. Falls in CHRs are associated with various conditions, such as gait or balance disorders, dizziness, confusion, and visual disorders [13, 15]. Furthermore, a fall might be the only symptom of acute medical illness in a geriatric patient [13]. An older British study found that around a third of all patients aged 80 + admitted to hospital due to medical illnesses had experienced a fall within seven days prior to admission, without the fall being the reason for hospital transfer [16]. This underlines that a ground-level fall might indicate an undetected acute illness in older adults.

On the other hand, an adverse event such as a fall might trigger the development of acute disease, e.g. pneumonia or delirium. A study showed that 8% of community-dwelling older adults treated for a traumatic injury (69% fall-related) and released from the ED experienced an in-hospital admission within the next 30 days, hereof 64.5% due to non-traumatic illnesses such as infections and congestive heart failure [17]. It is very difficult to distinguish whether the acute illness arose before or as a consequence of the fall that resulted in an ED visit.

Only little is known about the outcome for CHRs aged 65 + years following treat-and-release ED visits for traumatic events, which are often fall-related. We hypothesised that a traumatic event treat-and-release ED visit serves as an indicator for an upcoming acute hospital admission due to non-trauma-related conditions. In light of this, the present study aims to contribute to the discussion on preventing acute admissions of CHRs by:

1. Describing the resident’s traumatic event ED visits in terms of patterns for visits and primary discharge diagnoses.
2. Describing the frequency of subsequent admissions and treat-and-release revisits to the ED within 30 days of discharge from a treat-and-release visit, and assessing primary discharge diagnoses from subsequent admissions.
3. Analysing whether a treat-and-release ED visit for a traumatic event is associated with a higher 30-day admission rate compared to CHRs with no recent traumatic event ED visit.

## Methods

### Study design, population and terminology

This is a register-based cohort study. All CHRs living in Southern Jutland from 01 January 2018 to 31 December 2019 and aged 65 + years were eligible for the present study. We included all residents who experienced an acute ED visit due to a traumatic event within the two-year study period.

ED visits for traumatic events were divided into two categories by destination: Treat-and-release visits (duration ≤ 6 h) or admissions (duration > 6 h). The subgroup of residents who were discharged from a treat-and-release visit was observed for a maximum of 30 days regarding subsequent hospital admissions or treat-and-release revisits. Only the first hospital retransfer was included in the study.

If a resident had more traumatic event ED visits in the study period, only the first event was included in the assessment of baseline characteristics and in the regression analysis. In the assessment of patterns of traumatic event ED visits and hospital retransfer after treat-and-release visits, all events were included.

### Data sources

CHRs were identified through Care Home Data (in Danish: Plejehjemsdata), a new Danish national care home registry containing highly valid information on all Danish citizens living in care homes from 2014 to the present [18]. CHRs are identified with a civil registration number, which serves as a link to other national registers. Information on date of birth, sex, and date of death (if relevant) was obtained from the Danish Civil Registration System (founded in 1968), which contains general information on the entire Danish population [19]. Data on all contacts to the secondary health care system was assembled from the Danish National Patient Register, which contains information on all non-psychiatric hospital admissions since 1977 and all inpatient and outpatient activities in the entire secondary health care system since 1995 [20]. For every contact, one primary and optional secondary diagnoses are recorded according to the International Classification of Diseases (ICD-10). All acute somatic patients are registered with a reason for hospital referral: Either *disease, traumatic event, violence, self-harm, complications to former traumatic injuries, other*, or *unspecified*. The present study includes only hospital referrals where the primary reason for referral is *traumatic event*.

### Setting

In Denmark, citizens with permanent and substantial impairment of physical or mental function are given the opportunity to live in care homes. All Danish citizens can apply for care home residency, and local municipalities allocate residencies based on needs, not socioeconomic status [21]. All care homes operate under the Danish Consolidation Act on Social Services [21].

Southern Jutland is a geographical part of Denmark, comprising four municipalities with around 225,000 inhabitants living in both rural and urban areas. Population demographics are rather similar to the Danish population; citizens aged 65 + years accounted for 23.4% of the population in Southern Jutland and 19.6% of the Danish population in 2019 [22]. CHRs accounted for 0.67% of citizens living in Southern Jutland and 0.69% of all Danish citizens in 2019 [23]. The present study includes information from 2018 to 2019 on traumatic event ED visits by CHRs living in the 38 care homes in Southern Jutland, with approximately 1,600 long-term beds in total [24].

The Danish healthcare system is tax-funded and provides all citizens free and equal healthcare access. The EDs operate 24/7, and patients can be referred either through their primary care physician (PCP), by contacting the on-call PCP, or by calling the national emergency number 1-1-2 [25]. All acute hospital referrals due to traumatic events are initiated in the ED. From the ED, patients may be discharged within 6 h (termed treated and released throughout this article), admitted within the ED (for expected short admission < 48 h), or admitted to an in-hospital ward (for expected more prolonged admission > 48 h), both termed hospital admissions throughout this article.

### Data variables

#### Baseline characteristics

Residents were described in terms of sex and age at first traumatic event ED visit in the study period.

#### Traumatic event emergency department visits

We described all acute traumatic event referrals to the ED of CHRs in 2018–2019 in terms of day of the week, time of day, duration of stays, and reasons for visits. Reasons for visits were assessed using only the primary discharge diagnoses, and ICD-10 codes were categorised into subgroups within the ICD-10 chapters. Results are presented as total and stratified by destination (treated and released within 6 h or hospitalised). In-hospital mortality and mortality 30 days post-discharge are presented as total.

#### Traumatic event treat-and-release visits and subsequent hospital retransfers

For the subgroup of patients who were treated for traumatic events and released within 6 h, we evaluated the outcome 30 days post-discharge regarding new acute treat-and-release revisits to the ED (duration ≤ 6 h), subsequent acute hospital admissions (duration > 6 h), and reasons for admissions based on primary discharge diagnoses and hospital referral codes.

### Data analysis and statistical methods

Data are presented as total and proportions. However, duration of stay is measured as median with interquartile range (IQR), and age is presented as mean and standard deviation (SD).

The incidence of traumatic event ED visits was calculated based on resident-time at risk. The resident-time at risk is equivalent to the mean duration of care home stays in 2018–2019.

#### Primary outcome: traumatic event treat-and-release visits as an indicator of an impending acute hospital admission

We analysed whether a treat-and-release visit for a traumatic event acts as a warning sign for an upcoming hospital admission for CHRs. A treat-and-release ED visit due to a traumatic event was considered the exposure of interest, and we hypothesised that exposed residents had a higher rate of acute hospital admissions 30 days post-discharge when compared to the rate of admissions among unexposed residents (no recent traumatic event ED treat-and-release visit). We used a cause-specific Cox model [26] with time-varying exposure to model time to first admission within 30 days among CHRs with and without a treat-and-release visit for a traumatic event. We adjusted for age, sex, and duration of care home residency. We also adjusted for competing risks, with death as the competing event preventing CHRs from experiencing the event of interest (an acute admission). The proportional hazards assumption was tested using the Schoenfeld residuals.

No data was missing. All data for the present study was provided by the Danish Health Data Authority, and data was processed using Stata version 18.0.

### Ethics

The processing of personal data in the present study is notified to and approved by the Region of Southern Denmark and listed in the internal record (19/432,119) cf. Art 30 of The General Data Protection Regulation. According to Danish law, register-based studies do not require approval from an ethics committee or informed consent from the study participants [27].

## Results

### Participants and baseline characteristics

During 2018–2019, a total of 2,601 individuals aged 65 + resided in care homes in Southern Jutland. A full description of the background population of CHRs regarding morbidities, survival after care home admission, and acute hospitalisations in 2018–2019 can be found elsewhere [28]. Of the 2,601 CHRs, 507 individuals experienced at least one acute ED visit due to a traumatic event, and these patients compose the study population for the present study. Most patients were women (69.6%), and the mean age at the first traumatic event ED visit in the study period was 86.1 years (SD ± 7.4).

### Traumatic event emergency department visits

In the study period, 703 ED visits for traumatic events occurred. Residents were treated and released from the ED within 6 h in 506 cases (72.0%), while the remaining 197 visits (28.0%) resulted in acute hospital admission (either within the ED or in a hospital ward). The median duration of treat-and-release visits was 1.9 h (IQR 1.0–3.0 h). Median patient days for those admitted to hospital were 2.3 days (IQR 1.2–4.2 days). We found an annual incidence rate of 0.23 traumatic event ED visits per resident. Table 1 outlines the traumatic event ED visits of CHRs during 2018–2019.

**Table 1:** All traumatic event Emergency Department visits of care home residents in Southern Jutland in 2018–2019, stratified by destination (treated and released or hospitalised)

Traumatic event ED visits:		Treated and released (duration ≤ 6 h) *n* = 506 (72.0%)	Hospital admission (duration > 6 h) *n* = 197 (28.0%)	Total *n* = 703 (100%)
Day of visit				
	Weekday (Monday-Friday)	386 (76.3%)	148 (75.1%)	534 (76.0%)
	Weekend (Saturday-Sunday)	120 (23.7%)	49 (24.9%)	169 (24.0%)
Time of visit				
	Day-shift (08.00-15.59)	303 (59.9%)	88 (44.7%)	391 (55.6%)
	Evening (16.00-23.59)	154 (30.4%)	80 (40.6%)	234 (33.3%)
	Night (00.00-07.59)	49 (9.7%)	29 (14.7%)	78 (11.1%)
Diagnoses at discharge – most frequent groups of discharge diagnoses				
	Contusions and superficial injuries	169 (33.4%)	11 (5.9%)	180 (25.6%)
	Open wounds	93 (18.4%)	3 (1.5%)	96 (13.6%)
	Fractures in upper limbs	78 (15.4%)	8 (4.1%)	86 (12.2%)
	Fractures of the femur	2 (0.4%)	97 (49.2%)	99 (14.1%)
	Other lower limb fractures	15 (3.0%)	1 (0.5%)	16 (2.3%)
	Other fractures	25 (4.9%)	12 (6.1%)	37 (5.3%)
	Dislocations, sprains and strains	43 (8.5%)	4 (2.0%)	47 (6.7%)
	Intracranial injury	13 (2.6%)	8 (4.1%)	21 (3.0%)
	Other injuries	27 (5.3%)	13 (6.6%)	40 (5.7%)
	Medical observation and evaluation for suspected diseases and conditions	24 (4.7%)	10 (5.1%)	34 (4.8%)
	Other	17 (3.4%)	30 (15.2%)	47 (6.7%)

The most common three groups of discharge diagnoses from treat-and-release visits were contusions and superficial injuries, open wounds, and fractures in upper limbs, which comprised 67.2% of all discharge diagnoses. Femur fractures were the most dominant reason for hospitalisations, comprising 49.2% of all discharge diagnoses in cases where a hospital admission was needed. For a detailed description of all discharge diagnoses and the creation of sub-groups within the ICD-10 chapters, see Additional File 1.

In-hospital mortality was 2.3%. In comparison, 10.4% of all traumatic event visits resulted in death within 30 days of discharge, with 13 of 73 deaths occurring during a new acute hospital admission.

### Traumatic event treat-and-release visits and subsequent hospital retransfers

**Fig. 1:**
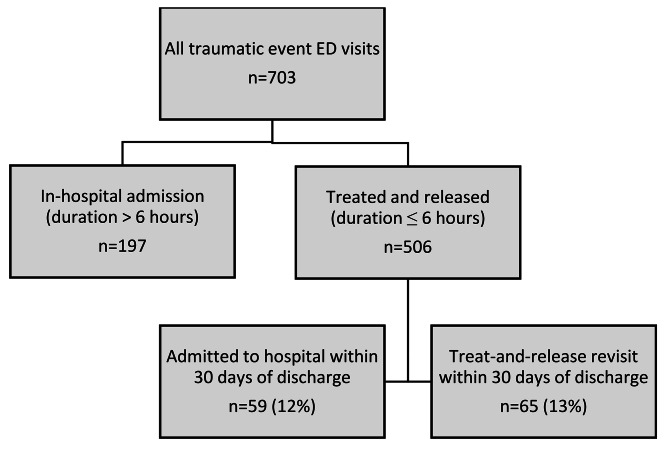
All traumatic event Emergency Department visits by care home residents in 2018–2019 in terms of destination (hospitalised or treated and released) and new hospital referrals within 30 days of discharge from a treat-and-release visit

In the subgroup of traumatic event ED visits where residents were treated and released from the ED within 6 h (*n* = 506), a total of 25% experienced a new acute ED transfer within 30 days of discharge, either resulting in a treat-and-release revisit (13%) or an admission (12%), as shown in Fig. 1. Of these new acute ED transfers, 56% were initiated within the first seven days post-discharge. Discharge diagnoses from the 59 subsequent hospital admissions within 30 days of a traumatic event treat-and-release visit were diverse and are shown in Table 2. Admissions due to injury (ICD-10 chapter XIX) comprised 25% (femur fractures 15%), diseases of the respiratory system (ICD-10 chapter X) comprised 17% (pneumonia 10%), and diseases of the circulatory system (ICD-10 chapter IX) comprised 13%. For a more complete description of all primary discharge diagnoses from subsequent hospital admissions within 30 days of a traumatic event treat-and-release visit, see Additional File 2.

**Table 2:** Showing all primary discharge diagnoses from subsequent admissions within 30 days of a treat-and-release Emergency Department visit due to a traumatic event

Chapter	Title	Total *n* (%)
I	Certain infectious and parasitic diseases	6 (10%)
IV	Endocrine, nutritional and metabolic diseases	1 (2%)
V	Mental and behavioural disorders	6 (10%)
IX	Diseases of the circulatory system	8 (13%)
X	Diseases of the respiratory system	10 (17%)
XI	Diseases of the digestive system	1 (2%)
XIV	Diseases of the genitourinary system	4 (7%)
XVIII	Symptoms, signs and abnormal clinical and laboratory findings, not elsewhere classified	5 (8%)
XIX	Injury, poisoning and certain other consequences of external causes	15 (25%)
XXI	Factors influencing health status and contact with health services	3 (5%)
	Total	59 (100%)

When investigating the reasons for renewed hospital referral in the 59 cases where a CHR experienced a subsequent hospital admission (duration > 6 h) within 30 days of a traumatic event treat-and-release visit, we observed that 10 (17%) were referred to hospital due to new traumatic events, and 13 (22%) were referred due to complications arising from previous traumatic injuries, see Table 3. Among the 13 subsequent admissions attributed to complications to previous traumatic injuries, six individuals were admitted due to fractures (five femur fractures and one pelvic fracture). In contrast, the remaining seven individuals were admitted for various conditions arising as complications to the preceding traumatic event. All three admissions with an unspecified reason for contact were initiated in the psychiatric ward, giving a total of 43 (73%) subsequent admissions due to non-injury conditions.

**Table 3:** Primary reasons for renewed acute hospital referral initiated within 30 days of an acute traumatic event treat-and-release emergency department visit of care home residents living in Southern Jutland in 2018–2019

Reason for referral, *n* (%)			
	Treat-and-release ED revisits (duration ≤ 6 h)	Subsequent admissions (duration > 6 h)	All new acute hospital referrals in total
Disease	18 (28%)	33 (56%)	51 (41%)
Traumatic event	27 (42%)	10 (17%)	37 (30%)
Complications to former traumatic injury	19 (29%)	13 (22%)	32 (26%)
Other	1 (1%)	0 (0%)	1 (1%)
Unspecified	0 (0%)	3 (5%)	3 (2%)
Total	65 (100%)	59 (100%)	124 (100%)

### Primary outcome: traumatic event treat-and-release visits as an indicator of an impending acute hospital admission

As shown in Table 4, a previous treat-and-release ED visit for a traumatic event was the largest predictor of hospital admission within 30 days, with a hazard ratio of 2.20 (95% CI: 1.52–3.17). Males had a higher hazard ratio of hospital admission of 1.73 (95% CI: 1.24–2.41). Age and duration of care home stays were no predictors of hospital admission.

**Table 4:** Fully adjusted hazard ratios for admission within 30 days of discharge from a treat-and-release Emergency Department visit due to traumatic events among care home residents

Covariate	Hazard ratio (95% CI)
Age	1.01 (0.99–1.03)
Males	**1.73 (1.24–2.41)**
Duration of care home residency	1.00 (1.00–1.00)
Exposure (treat-and-release ED visit)	**2.20 (1.52–3.17)**

Significant findings are in bold.

## Discussion

### Summary

The hazard of experiencing an acute hospital admission was 2.20 times higher among CHRs with a recent ED treat-and-release visit due to a traumatic event when compared to the background population of CHRs with no recent traumatic event visits. Almost three-fourths of admissions arising after discharge from treat-and-release traumatic event ED visits were caused by medical issues, supporting our initial hypothesis that traumatic event ED visits serve as indicators for upcoming acute hospital admission due to non-trauma-related conditions.

Regarding the reasons for hospital admissions within 30 days of a traumatic event ED treat-and-release visit, 17% were referred to hospital due to new traumatic events, while 22% of referrals were attributed to complications arising from previous traumatic injuries (medical or surgical).

Most traumatic event ED visits occurred on weekdays (76.0%) during day shifts (55.6%). In the majority of cases, patients were treated and released within 6 h (72.0%), while the remaining required a more prolonged hospital admission. Treat-and-release visits were often due to minor injuries such as contusions and superficial injuries, open wounds, and dislocations, sprains and strains, accounting for 60.3% of all discharge diagnoses.

In-hospital mortality for traumatic event ED visits was 2.3%, while in 10.4% of events, the CHRs died within 30 days of discharge. When investigating the 506 events where residents were treated and released within 6 h, 25% returned to hospital within 30 days of discharge from the ED, hereof more than half within the first seven days post-discharge.

### Traumatic event emergency department visits

Our findings regarding the course of treatment in traumatic event visits to the ED are in accordance with other recent studies on CHRs presenting to the ED with injuries/trauma, where around two-thirds of patients are managed within the ED with no need for further admission [6, 29]. CHRs seen in the ED due to traumatic events thereby have a significantly lower incidence of hospitalisation from the ED than CHRs presenting with non-traumatic conditions [4, 29], as many injuries are minor and can be treated without the need for hospitalisation. Table 1 confirms that CHRs were often treated for minor injuries such as contusions and superficial injuries, open wounds, and dislocations, strains and sprains, and released within 6 h. However, we still found a high prevalence of subsequent hospital admissions and treat-and-release ED revisits, indicating a period of increased vulnerability after the initial ED visit.

### Mortality

A Norwegian study on acute hospital admissions of CHRs found a similar in-hospital mortality rate (4.1%) and 30-day mortality rate (14.4%) when investigating the outcome for CHRs admitted due to *injury, poisoning and certain other consequences of external causes* (ICD-10 chapter XIX) [2]. When evaluating admissions of CHRs due to all causes in the Norwegian study, overall in-hospital mortality was 16%, and 29% died within the first 30 days after discharge. The higher mortality associated with all-cause admissions is within range of the results of a systematic review [4]. Thus, CHRs admitted due to non-traumatic conditions have a higher mortality, both in-hospital and post-discharge, when compared to CHRs admitted for trauma/injuries.

### Traumatic event treat-and-release visits and subsequent hospital retransfers

When focussing on residents who were treated and released from the ED within 6 h, we found that 42% (*n* = 27) of individuals who experienced a new treat-and-release visit and 17% (*n* = 10) of individuals with a subsequent hospital admission were referred to the ED due to a new traumatic event (Table 2). An American study on falls among community-dwelling older adults showed a lower rate of 30-day readmissions of 11.3%, and 15% of readmissions were due to a recurrent fall [14]. Notably, the rate of 30-day readmissions for new fall-related injuries was approximately 4.5 times higher among fallers than that observed for non-fall trauma patients (3.5%). This indicates an ongoing risk of falls, which calls for preventive measures to reduce the likelihood of recurrent incidents.

It is important to acknowledge that some subsequent admissions due to complications in our study can occur because patients are recalled to the hospital when a review of the X-rays taken during the initial contact reveals a previously undetected fracture. This situation can exaggerate the perceived risk of admission following a treat-and-release ED visit. It is possible to argue that more comprehensive initial assessments might have led to their admission during the first visit, thereby avoiding the subsequent admission. Conversely, some of the six patients admitted due to fractures may be admitted due to persistent pain, prompting new X-rays that reveal the fracture. On the other hand, one might argue that the subsequent admission due to a urinary tract infection or pneumonia could have been prevented with a more thorough assessment of the CHR’s health status at the initial ED visit.

### Traumatic event treat-and-release visits as an indicator of an impending acute hospital admission

Although CHRs are generally often admitted acutely to hospital, there is a remarkably increased rate of acute admissions following a treat-and-release visit for a traumatic event, mainly due to disease or medical complications to former traumatic injuries. Our discovery that 73% of subsequent admissions stem from a wide range of non-injury-related medical issues is a key finding in our study, and it confirms our initial hypothesis: The event (often a ground-level fall) that resulted in the injury might indicate an undetected disease. A recent study on community-dwelling older adults in the USA who were treated for injuries and released from the ED showed a readmission rate of 7.9% within 30 days, hereof 64.5% due to non-trauma [17]. This highlights that a traumatic event such as a fall can indicate an incipient acute illness such as an infection or a deterioration of chronic disease. The ED physician focuses on the consequences of the fall (e.g., the wound or suspected fracture) instead of the causes of the fall, which might lead to any missed diagnoses. Upon the initial traumatic event visit, a more thorough assessment of the CHRs’ health (e.g., vital signs, blood samples with focus infection and fluid balance, and urine sample) might help prevent some subsequent admissions, even when the ED visit is for only minor injuries.

On the other hand, it is conceivable that a fall can lead to the development of acute illness, such as pneumonia occurring after contusions against the thorax, delirium due to pain or opioid use, or dehydration or embolism due to inactivity. The high admission rate might indicate an increased frailty after an acute treat-and-release ED visit, where residents are more prone to diseases. The retrospective register-based design of the present study makes it difficult to conclude whether the acute illness resulted in the fall or arose as a consequence of the fall. A thorough assessment of journals from hospitals, PCPs and care homes at the specific time up to and after treat-and-release ED visits could shed some light on this question.

### Strengths and weaknesses

The present study contains a complete and relatively large cohort of all CHRs living in Southern Jutland. Data are enriched with highly valid information on all hospital contacts from Danish national health registers [30]. These significant strengths enable us to produce a high-quality register-based study. However, it is important to recognise that our understanding is limited by the data collected in the registries used in the present study. Register-based data may not capture nuanced details or factors that are essential for a comprehensive understanding of the subject. Furthermore, it cannot be guaranteed that all traumatic events have been recorded with this as the reason for hospital referral. In theory, actual traumatic events incorrectly coded with *disease* or *complications to former traumatic injuries* or *other* as the primary reason for hospital transfer are excluded from the present study, leading to an underestimation of the prevalence of traumatic events among CHRs.

Our findings not only shed light on patterns of subsequent hospital admissions among CHRs following traumatic event ED visits but also hold significant implications for enhancing care transition and continuity of care in this population. Our study underscores the importance of comprehensive health assessments during initial ED visits to mitigate the risk of subsequent acute hospital admissions.

In line with this, it is essential to consider the concept of extended treatment responsibility, as practised in Denmark since 2023, where hospitals retain responsibility for patient care for a designated period post-discharge. This model aims to ensure a smoother transition and continuity of care for patients, providing support and guidance for healthcare professionals during the post-discharge period [31]. This can be crucial for optimising patient outcomes and experiences and to avoid unnecessary readmissions.

Future research should explore strategies to improve care transition processes and ensure seamless continuity of care for CHRs. This could involve engaging CHRs and their families to gain insights into their healthcare needs, preferences and experiences during care transitions. Interventional studies with a more comprehensive assessment of CHRs’ health status upon traumatic event ED visits are needed to evaluate the potential for reducing subsequent hospital admissions.

## Conclusion

Traumatic event treat-and-release ED visits among care home residents serve as an indicator for subsequent hospital admissions. This underlines the importance of considering a more comprehensive evaluation of care home residents when they seek treatment at the emergency department, even for minor injuries.

### Electronic supplementary material

Below is the link to the electronic supplementary material.

Supplementary Material 1

Supplementary Material 2

## Data Availability

The data that support the findings of this study are available from the Danish Health Data Authority but restrictions apply to the availability of these data, which were used under licens for the current study, and so are not publicly available. Data are however available from the corresponding author upon a reasonable request and with permission of the Danish Health Data Authority.

## Abbreviations

CHRs: Care home residents
CI: Confidence interval
ED: Emergency department
ICD-10 codes: International Classification of Diseases
PCP: Primary care physician
SD: Standard deviation
