# Supplementary material for: Analysis of traumatic event emergency department visits among care home residents aged 65 + years in Southern Jutland, Denmark: implications for comprehensive care and subsequent hospital admissions - a register-based cohort study
Source: BMC Geriatr. 2024 May 28;24:465. doi: 10.1186/s12877-024-05092-0 (PMC11134667; doi:10.1186/s12877-024-05092-0)
Supplement: Supplementary file 1 — Supplementary Material 1 [file 12877_2024_5092_MOESM1_ESM.docx]

**Supplementary Table 2: All primary discharge diagnoses from acute hospitalisations of care home residents within 30 days of a treat-and-release visit for injuries in Southern Jutland in 2018-2019**

| **Chapter** | **Title** | **Subcategories** | **ICD-10 codes** | **Total n (%)** | |
| --- | --- | --- | --- | --- | --- |
|  |  |  |  |  | **Hereof** |
| **I** | **Certain infectious and parasitic diseases** |  | **A00-B99** | **6 (10%)** | |
| **IV** | **Endocrine, nutritional and metabolic diseases** |  | **E00-E90** | **1 (2%)** | |
| **V** | **Mental and behavioural disorders** |  | **F00-F99** | **6 (10%)** | |
| **IX** | **Diseases of the circulatory system** |  | **I00-I99** | **8 (13%)** | |
|  |  | Pulmonary embolism | I26 |  | 3 (5%) |
|  |  | Other |  |  | 5 (8%) |
| **X** | **Diseases of the respiratory system** |  | **J00-J99** | **10 (17%)** | |
|  |  | Pneumonia | J13-J18 |  | 6 (10%) |
|  |  | Pneumonitis due to solids and liquids | J69 |  | 1 (2%) |
|  |  | Respiratory failure, not elsewhere classified | J96 |  | 3 (5%) |
| **XI** | **Diseases of the digestive system** |  | **K00-K93** | **1 (2%)** | |
| **XIV** | **Diseases of the genitourinary system** |  | **N00-N99** | **4 (7%)** | |
|  |  | Urinary tract infection | N30, N390 |  | 2 (3%) |
|  |  | Other |  |  | 2 (3%) |
| **XVIII** | **Symptoms, signs and abnormal clinical and laboratory findings, not elsewhere classified** |  | **R00-R99** | **5 (8%)** | |
| **XIX** | **Injury, poisoning and certain other consequences of external causes** |  | **S00-T98** | **15 (25%)** | |
|  |  | Fractures of the femur | S720-S729 |  | 9 (15%) |
|  |  | Other fractures | S02, S098B, S12, S22, S32, S42, S52, S62, S820-S829, S92 |  | 1 (2%) |
|  |  | Intracranial injury | S06 |  | 2 (3%) |
|  |  | Other |  |  | 3 (5%) |
| **XXI** | **Factors influencing health status and contact with health services** |  | **Z00-Z99** | **3 (5%)** | |
|  | Total |  |  | 59 (100%) | |
