# Supplementary material for: Analysis of traumatic event emergency department visits among care home residents aged 65 + years in Southern Jutland, Denmark: implications for comprehensive care and subsequent hospital admissions - a register-based cohort study
Source: BMC Geriatr. 2024 May 28;24:465. doi: 10.1186/s12877-024-05092-0 (PMC11134667; doi:10.1186/s12877-024-05092-0)
Supplement: Supplementary file 2 — Supplementary Material 2 [file 12877_2024_5092_MOESM2_ESM.docx]

**Supplementary Table 1: All primary discharge diagnoses from acute emergency department visits for traumatic injuries among care home residents living in Southern Jutland in 2018-2019**

| **Chapter** | **Title** | **Subcategories** | **ICD-10 codes** | **Total n (%)** | |
| --- | --- | --- | --- | --- | --- |
|  |  |  |  |  | **Hereof** |
| **I** | **Certain infectious and parasitic diseases** |  | **A00-B99** | **5 (0.7%)** | |
| **III** | **Diseases of the blood and bloodforming organs and certain disorders involving the immune mechanism** |  | **D50-D89** | **1 (0.1%)** | |
| **IV** | **Endocrine, nutritional and metabolic diseases** |  | **E00-E90** | **2 (0.3%)** | |
| **V** | **Mental and behavioural disorders** |  | **F00-F99** | **2 (0.3%)** | |
| **IX** | **Diseases of the circulatory system** |  | **I00-I99** | **2 (0.3%)** | |
| **X** | **Diseases of the respiratory system** |  | **J00-J99** | **8 (1.1%)** | |
|  |  | Pneumonia | J13-J18 |  | 6 (0.8%) |
|  |  | Respiratory failure, not elsewhere classified | J96 |  | 2 (0.3%) |
| **XIII** | **Diseases of the musculoskeletal system and connective tissue** |  | **M00-M99** | **4 (0.6%)** | |
| **XIV** | **Diseases of the genitourinary system** |  | **N00-N99** | **1 (0.1%)** | |
| **XVIII** | **Symptoms, signs and abnormal clinical and laboratory findings, not elsewhere classified** |  | **R00-R99** | **9 (1.3%)** | |
|  |  | Difficulty in walking, not elsewhere classified | R262 |  | 4 (0.6%) |
|  |  | Other |  |  | 5 (0.7%) |
| **XIX** | **Injury, poisoning and certain other consequences of external causes** |  | **S00-T98** | **622 (88.5%)** | |
|  |  | Contusions and superficial injuries | S00, S10, S20, S30, S40, S50, S60, S70, S80, S90 |  | 180 (25.6%) |
|  |  | Open wounds | S01, S11, S21, S31, S41, S51, S61, S71, S81, S91 |  | 96 (13.6%) |
|  |  | Dislocations, sprains and strains | S03, S13, S23, S33, S43, S53, D63, S73, S83, S93 |  | 47 (6.7%) |
|  |  | Intracranial injury | S06 |  | 21 (3.0%) |
|  |  | Fractures in upper limbs | S42, S52, S62 |  | 86 (12.2%) |
|  |  | Fractures of the femur | S720-S729 |  | 99 (14.1%) |
|  |  | Other lower limb fractures | S820-S829  S92 |  | 16 (2.3%) |
|  |  | Other fractures | S02, S12, S22, S32 |  | 37 (5.3%) |
|  |  | Other |  |  | 40 (5.7%) |
| **XXI** | **Factors influencing health status and contact with health services** |  | **Z00-Z99** | **47 (6.7%)** | |
|  |  | Medical observation and evaluation for suspected diseases and conditions, ruled out. | Z03 |  | 34 (4.8%) |
|  |  | Other |  |  | 13 (1.8%) |
|  | Total |  |  | 703 (100.0%) | |
